# Exploring the Glucose Fluxotype of the *E. coli* y-ome Using High-Resolution Fluxomics

**DOI:** 10.3390/metabo11050271

**Published:** 2021-04-26

**Authors:** Cécilia Bergès, Edern Cahoreau, Pierre Millard, Brice Enjalbert, Mickael Dinclaux, Maud Heuillet, Hanna Kulyk, Lara Gales, Noémie Butin, Maxime Chazalviel, Tony Palama, Matthieu Guionnet, Sergueï Sokol, Lindsay Peyriga, Floriant Bellvert, Stéphanie Heux, Jean-Charles Portais

**Affiliations:** 1 Toulouse Biotechnology Institute (TBI), Université de Toulouse, CNRS, INRAE, INSA, 31077 Toulouse, France; berges@insa-toulouse.fr (C.B.); cahoreau@insa-toulouse.fr (E.C.); millard@insa-toulouse.fr (P.M.); brice.enjalbert@insa-toulouse.fr (B.E.); mickael.dinclaux@inrae.fr (M.D.); maud.heuillet@gmail.com (M.H.); hbarbier@insa-toulouse.fr (H.K.); lara.gales@insa-toulouse.fr (L.G.); butin@insa-toulouse.fr (N.B.); tony.palama@univ-paris13.fr (T.P.); matthieu.guionnet@univ-tlse2.fr (M.G.); sokol@insa-toulouse.fr (S.S.); peyriga@insa-toulouse.fr (L.P.); bellvert@insa-toulouse.fr (F.B.); heux@insa-toulouse.fr (S.H.); 2 MetaToul-MetaboHUB, National Infrastructure of Metabolomics & Fluxomics (ANR-11-INBS-0010), 31077 Toulouse, France; 3 RESTORE, Université de Toulouse, Inserm U1031, CNRS 5070, UPS, EFS, 31100 Toulouse, France; 4 Toxalim (Research Centre in Food Toxicology), UMR1331, Université de Toulouse, INRAE, ENVT, INP-Purpan, UPS, 31300 Toulouse, France; maxime.chazalviel@gmail.com

**Keywords:** high-resolution fluxotyping, fluxomics, high throughput, y-ome phenotyping, *E. coli*

## Abstract

We have developed a robust workflow to measure high-resolution fluxotypes (metabolic flux phenotypes) for large strain libraries under fully controlled growth conditions. This was achieved by optimizing and automating the whole high-throughput fluxomics process and integrating all relevant software tools. This workflow allowed us to obtain highly detailed maps of carbon fluxes in the central carbon metabolism in a fully automated manner. It was applied to investigate the glucose fluxotypes of 180 *Escherichia coli* strains deleted for y-genes. Since the products of these y-genes potentially play a role in a variety of metabolic processes, the experiments were designed to be agnostic as to their potential metabolic impact. The obtained data highlight the robustness of *E. coli*’s central metabolism to y-gene deletion. For two y-genes, deletion resulted in significant changes in carbon and energy fluxes, demonstrating the involvement of the corresponding y-gene products in metabolic function or regulation. This work also introduces novel metrics to measure the actual scope and quality of high-throughput fluxomics investigations.

## 1. Introduction

Despite the progress that has been made in sequencing technology and genome annotation, a non-negligible percentage of genes remains uncharacterized [1]. Even for very well-known organisms, such as the bacterium *E. coli*, 35–40% of genes (the ‘y-ome’) are of unknown function [2,3]. The products of these genes (y-genes) are likely to have highly diverse functions among the various cellular processes. Metabolism is the basic process that sustains all the energetic and biosynthetic needs of living organisms to survive and grow. Not surprisingly, attempts have been made to investigate the potential role of the y-genes in metabolic processes [4,5]. Accordingly, several high throughput (HT) methods have recently been developed to explore the potential metabolic function of y-genes. This includes the incubation of purified proteins with metabolite cocktails, the identification by mass spectrometry of metabolites with varying abundances [6] and the application of untargeted metabolomics to map gene-metabolite interactions [5]. 

Alongside studies aimed at directly identifying gene product function, an alternative strategy for studying the role of y-genes consists of detailing the phenotypic consequences of a loss-of-function mutation or a gene deletion [7,8]. Together with the generation of mutant libraries, such as the Keio *E. coli* mutant collection [9], which facilitates genome-wide investigations, this approach has been used for large-scale investigations of gene essentiality [9,10,11,12] and of molecular [13], morphological [14] and fitness [15] phenotypes in bacteria. Metabolic phenotyping approaches have also been developed in which comparative metabolomics is applied to reveal gene functions in yeast [4].

Metabolic phenotypes are most accurately revealed by fluxomics, which aims to measure the actual rates of biochemical reactions in metabolic networks [16]. Fluxomics measures the actual output of the integrated response of the gene-protein-metabolite interaction network [17] and provides direct access to the cellular phenotype in a quantitative manner [18]. It has, thereby, become a major tool in comprehensive investigations of cellular metabolism in many fields, ranging from biotechnology [19] to the medical sciences [20]. The measurement of metabolic fluxes is based on ^13^C-labelling experiments coupled with detailed mathematical models of metabolism (^13^C-fluxomics). This is a complex and tedious process involving many steps [16,21] and requiring significant expertise in data collection and interpretation. Obtaining the detailed flux information on hundreds of strains needed to explore the *E. coli* y-ome is a motivating factor in improving the current HT fluxomics workflows [22].

In this work, we developed a robust workflow, in order to obtain high-resolution fluxotypes under fully controlled growth conditions for large strain libraries. A fluxotype is defined here as the particular distribution of metabolic fluxes measured for a given strain under given physiological conditions. Resolution refers to the level of flux information that can be generated and is high when a significant number of fluxes can be measured [21,23,24]. This workflow was applied to investigate the fluxotypes of 180 *E. coli* strains that are deleted for y-genes and grown on glucose as sole carbon source. The data show that the central metabolism of *E. coli* is highly robust to y-gene deletion. For two y-genes, deletion resulted in significant alterations of metabolic fluxes, pointing to the role of the corresponding y-gene products in metabolic function and/or regulation. 

## 2. Results

### 2.1. Selection of E. coli y-ome Strains

**Figure 1:**
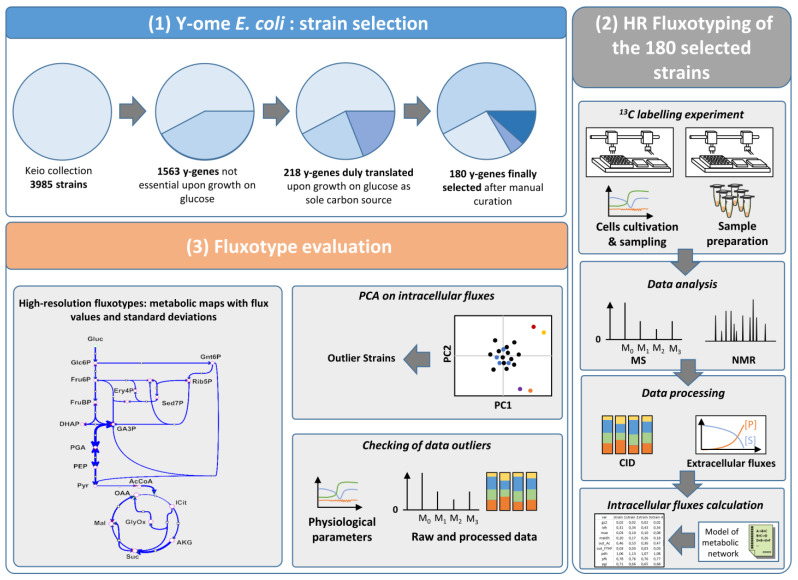
Strategy for high-resolution fluxotyping of the *E. coli* y-ome. Selection of *E. coli* y-genes (**1**) 180 y-genes were selected based on their expression on glucose as sole carbon source. High-resolution Fluxotyping (**2**) of 180 mutants with single-deletion of a selected y-gene. Cell cultivation, metabolite harvesting and sample preparation are performed by robotic systems. Quantification of medium compounds and isotopic profiling of metabolites by NMR and MS. Fluxes are calculated using a model of *E. coli* central carbon metabolism. Fluxotype evaluation (**3**) Metabolic fluxes are calculated for all strains (high-resolution fluxotypes) and compared by statistical analysis to identify altered fluxotypes. Fluxotype outliers are checked against culture data to ascertain that outlying flux values are not due to a ^13^C-labelling problem. Metabolites: Gluc.: Glucose, Glc6P: glucose-6-phopshate, Fru6P: fructose-6-phosphate, FruBP: fructose-bisphophate, DHAP: dihydroxyacetone-phosphate, GA3P: glyceraldehyde-3-phosphate, PGA: 3-phosphoglyceric acid, PEP: phosphoenolpyruvate, Pyr: pyruvate, AcCoA: acetylCoa, OAA: oxaloacetic acid, ICit: Isocitrate, AKG: alpha-ketoglutaric acid, Suc: succinate, Mal: malate, GlyOx: glyoxylate, Gnt6P: gluconate-6-phosphate, Rib5P: ribose-5-phosphate, Ery4P: erythrose-4-phosphate, Sed7P: sedoheptulose-7-phosphate.

Based on the aim of investigating the metabolic phenotypes of the *E. coli* y-ome during growth on glucose as sole carbon source, we first selected y-genes as follows (Figure 1):

(i) We first considered all genes with a single-deletion mutant in the Keio mutant library [9]. This library contained single-gene deletion mutants able to grow on glucose, meaning the mutated genes are not essential for growth on glucose as sole carbon source. The Keio collection contains 3985 mutants.
(ii) We, then, selected the genes in the Keio collection lacking evidence of function. This represented a total of 1563 y-genes
(iii) We verified that each y-gene was duly expressed and translated during growth on glucose as the sole carbon source. This selection step was based on the extensive proteomic investigation performed by Schmidt et al., in 2016 [25]. These authors measured the functional expression of 55% *E. coli* genes (>2300 genes) by quantitative proteomics in 22 experimental conditions, including growth in minimal medium with glucose as carbon source. Among the y-genes identified in step 2, we further selected the 218 y-genes that were experimentally shown to be translated under these conditions.
(iv) The y-gene status-i.e., the lack of annotation or of experimental evidence of function-was manually verified in two complementary databases, namely Biocyc (https://biocyc.org (accessed on 12 April 2019)) and Uniprot (https://www.uniprot.org (accessed on 12 April 2019)).

This process yielded a group of 180 y-genes with unknown or unclear function, dispensable, but duly expressed in a medium with glucose as sole carbon source (Supplementary data 1). The corresponding single deletion mutants were obtained from the Keio collection and considered for further metabolic investigations, which were designed to optimize the measurement of growth parameters and fluxotypes across hundreds of *E. coli* mutants (Figure 1). Fluxotypes can be visualized using a so-called flux map, a graphical representation of the metabolic network showing the flux values for the various reactions or pathways.

### 2.2. Integrated Workflow for High-Throughput Collection of High-Resolution Fluxotypes

To measure the fluxotypes of the 180 y-gene deletion mutants, we first built an HT platform enabling fully automated, parallelized measurements of metabolic fluxes under fully controlled conditions at a throughput consistent with the investigation of hundreds of experimental conditions. A typical ^13^C-fluxomics workflow involves a combination of several complex experimental and computational steps, which were improved and optimized here to meet the needs of the y-ome investigation (Figure 1). The final setup included (i) two automated robotic platforms, one for parallelized growth, ^13^C-labelling experiments and sample collection, and the other for sample preparation, (ii) optimized NMR and MS analytical methods for measuring metabolite concentrations and labeling patterns, (iii) flux calculation, (iv) statistical analysis, and (v) a series of software programs to store, manage, and process the data and meta-data generated all through the process. 

### 2.3. Design of Fluxomics Experiments

Very little is known about the phenotypic and metabolic behavior of y-gene–deleted strains, hence, an important objective of the setup was to avoid presuppositions about the metabolic pathways potentially impacted by the lack of the y-gene product. This was achieved by using a generic model of *E. coli* metabolism for the flux investigations, containing all central carbon metabolism pathways (Supplementary data 3). The model contained 94 fluxes in total, representing central pathways, biosynthetic processes, and transport reactions, and 49 metabolites. The isotopic composition of the label input in the ^13^C-labelling experiments was optimized using the software IsoDesign [26]. Based on, (i) the network topology, (ii) the isotopic data collected in the study (i.e., isotopologue distributions of proteinogenic amino-acids), and (iii) the objective of resolving the maximum number of fluxes across the whole metabolic network, the best label composition was determined to be a mixture of 80% [1-^13^C]-glucose + 20% [U-^13^C]-glucose. This result is consistent with those of previous investigations of the entire central carbon metabolism of *E. coli* [16]. All labeling experiments, reported in this study, were performed in minimal M9 medium with the above mixture as sole carbon source. Details of the conditions used to apply the workflow to all the investigations, reported here, are given in material and methods section. Briefly, cells were inoculated at 10^8^ cell/mL and their growth was monitored by optical density under temperature, pH and pO_2_ control. Medium samples were collected throughout the growth process and analyzed by ^1^H-NMR to measure the rates of substrate uptake and product release. When an Optical Density (OD_600nm_) of 1.2-corresponding to mid-exponential growth under these conditions-was reached, the biomass was automatically sampled, hydrolyzed, and the labeling patterns of proteinogenic amino acids were analyzed by LC-HRMS. After correction for naturally occurring isotopes [27,28], the carbon isotopologue distributions (CIDs) of 16 amino acids were measured and used for flux calculations. Biosynthetic fluxes (i.e., precursor requirements for growth) were calculated based on the molecular composition of *E. coli* [29]. Intracellular fluxes were calculated by fitting the metabolic model described above with amino acid CIDs, biosynthetic fluxes, and extracellular fluxes, using the software influx_si [30]. The confidence intervals on calculated fluxes were estimated by Monte Carlo sensitivity analysis using the same software. All investigations were performed on the robotic system, which allowed 48 experiments to be run in parallel, yielding 48 flux maps per run, each containing 94 fluxes, measured under fully controlled physiological conditions.

### 2.4. High-Resolution Fluxotyping Workflow Validation

**Figure 2:**
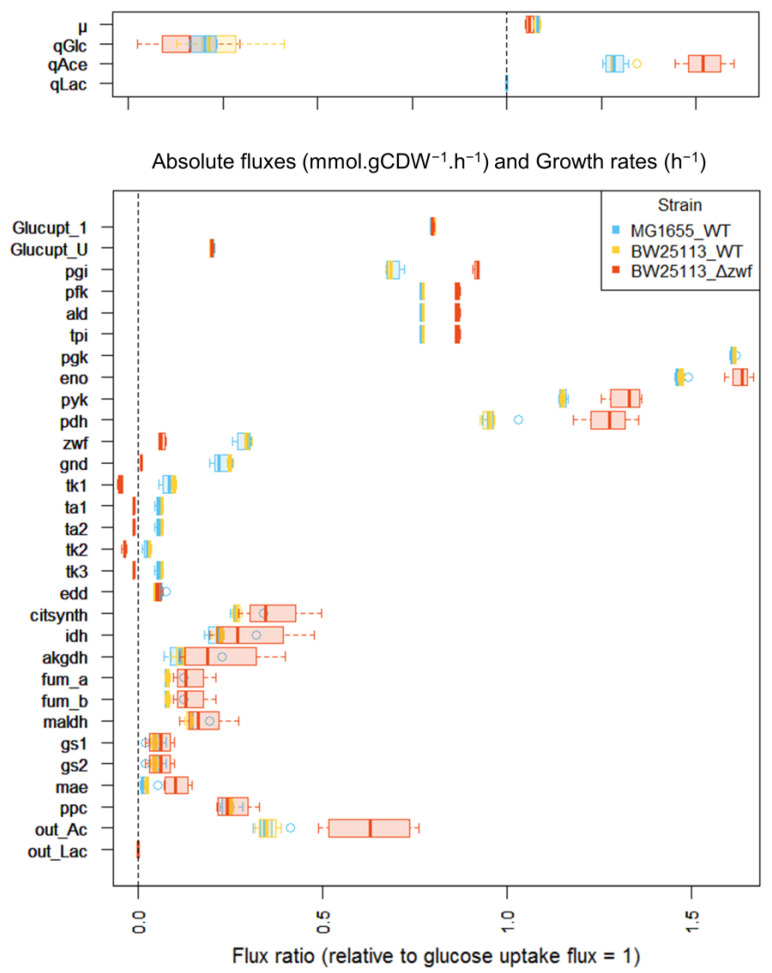
Metabolic fluxes measured for *E. coli* WT strains and a *Δ*zwf mutant strain. Top: experimentally measured fluxes (expressed in mmol·gDW^−1^·h^−1^) and growth rate (expressed in h^−1^). Physiological measurements: µ, growth rate; qGlc, glucose specific consumption rate; qAce, acetate specific production rate; qLac, lactate specific production rate. Bottom: Intracellular fluxes calculated from the ^13^C-labelling data; fluxes are expressed relative to the glucose uptake rate set arbitrarily to 1 for each strain. Boxplot produced with the software R representing median fluxes (bold line) in boxes representing the interquartile range (quartile 1–quartile 3). The whiskers extend to the most extreme data point that is no more than 1.5 times the interquartile range from the box. Net fluxes: Glucupt_1 and Glucupt_U: [1-^13^C]-glucose and [1-^13^C]-glucose importation (PTS system), pgi: glucose-6-phosphate isomerase, pfk: phosphofructokinase, ald: fructose-bisphosphate aldolase, tpi: triose-phosphate isomerase, pgk: phosphoglycerate kinase, eno: enolase, pyk: pyruvate kinase, pdh: pyruvate dehydrogenase, zwf: glucose 6-phosphate dehydrogenase, gnd: phosphogluconate dehydrogenase, tk1: half-reaction transketolase (1), ta1: half-reaction transaldolase (1), ta2: half-reaction transaldolase (2), tk2: half-reaction transketolase (2), tk3: tk2: half-reaction transketolase (3), edd: Entner-Doudoroff enzymes: equivalent to 6-phosphogluconate dehydratase and 2-keto-3deoxy-6phosphogluconate (KDPG) aldolase, citsynth: citrate synthase, idh: isocitrate dehydrogenase, akgdh: alpha-ketoglutarate dehydrogenase, fum_a and fum_b: fumarase, maldh: malate dehydrogenase, gs1: isocitrate lyase, gs2: malate synthase, mae: malic enzyme, ppc: equivalent to pep carboxylase (forward flux) and to pep carboxykinase (reverse flux), out_Ac: acetate output (equivalent to acetate kinase).

Prior to starting the investigation of the *E. coli* y-ome, we evaluated the workflow by performing several fluxomics experiments with known *E. coli* strains. Two wild-type (WT) strains (*K-12 MG1655* and *BW25113)* and one mutant strain (*BW25113 Δzwf*), with known and significant metabolic flux alterations, were considered to evaluate the biological relevance of the data collected with the integrated HT workflow. The data collected for the three strains were highly reproducible between replicates. The median relative standard deviation (RSD) of the central metabolic fluxes was 11%, 3.7% and 22% between 5 (*K-12 MG1655)*, 4 (*BW25113),* and 5 (*BW25113 Δzwf*) biological replicates, respectively. The macro-kinetic parameters (i.e., growth rate, glucose uptake, acetate production) and flux values collected for the two WT strains (Figure 2) were closely consistent with previous measurements on these strains, highlighting the consistency of the data collected with the integrated HT fluxomics workflow [22,31,32,33]. The deletion of *zwf* had little impact on the growth rate but led to a significant redistribution of metabolic fluxes to compensate for the impairment of the oxidative branch of the pentose phosphate pathway (PPP). The observed changes in metabolic fluxes in this strain were qualitatively and quantitatively consistent with previous reports [22,31,34,35]. The developed workflow’s ability to reliably measure the growth and fluxotypes of these strains clearly demonstrates the consistency of the entire process.

### 2.5. High-Resolution Glucose Fluxotyping of 180 Selected Y-Gene Mutant Strains

The 180 strains deleted for the selected y-genes were isolated from the Keio strain collection, and their growth profiles and fluxotypes were measured in minimal medium with glucose as sole carbon source. The WT and *Δzwf* strains were included in each robotic run as reference strains to check the inter-batch consistency of growth parameters and flux data. In total, 198 cultures were performed from which 1074 OD_600_ points were measured and used for the calculation of growth rates and biomass yields; 1248 samples were collected (1050 filtrates and 198 cell pellets), from which 591 physiological data, i.e., rates of compound consumption or production-were extracted. Further, more than 20,000 isotopic data measured (100 carbon isotopologues from 16 different metabolites by MS analyses and 6 positional labelling profiles for 2 metabolites by NMR for each cultivation). Finally, a cumulated total of 18,612 fluxes were calculated (94 fluxes for each culture). The complete experimental process was completed within 786 h, and required only 194 h, i.e., about 1 h per measured fluxotype-of human intervention or supervision. These values emphasize the practical benefit of the established workflow.

**Figure 3:**
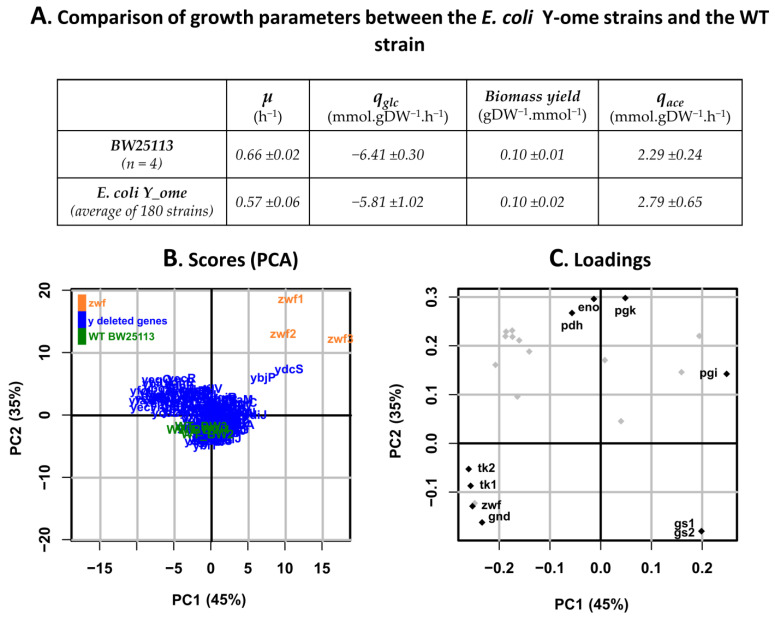
Comparison of the growth parameters and fluxotypes of y-ome strains with those of their parental strain. (**A**) Comparison of growth parameters between y-ome strains and the BW25113 WT strain (*µ*: growth rate, *q_glc_*: glucose consumption rate, *Biomass yield*, *q_ace_*: acetate production rate). (**B**) PCA plots showing the strains with the most different fluxotypes across the y-ome. PC1 and PC2 represent Principal Component 1 and 2, explaining the largest variance between strains according to their flux values-the percentage of variance explained for each principal component is indicated into brackets [36], (**C**) PCA loading plot with the most impacted fluxes indicated.

The data collected for the reference strains, i.e., WT and *Δzwf* strains-in the various runs of the robotic platform did not differ significantly from the data collected in the validation stage. On average, the strains deleted for y-genes grew slower than WT strains but had similar biomass yields (Figure 3A). The rates of glucose consumption and acetate production, measured from exometabolome data, were consistent with these observations, i.e., no significant difference overall. Principal component analysis (PCA) was used to explore the distribution of intracellular flux data across the y-ome (Figure 3B,C). For a large majority of mutant strains (in blue), the flux data-expressed relative to the glucose uptake rate-clustered with those of the WT strains (in green). This result indicates that, for most of the y-genes, deletion has a limited impact on the distribution of intracellular metabolic fluxes. The complete dataset is provided in Supplementary data 4.

Three mutant strains did not cluster with the WT strains (Figure 3B). One of these was the *Δzwf* strain, demonstrating the value of PCA to discriminate strains based on metabolic fluxes. Two other y-gene mutants, namely *ΔybjP* and *ΔydcS*, also had significantly different fluxotypes from those of the WT strains and other y-gene mutants. Loading plots (Figure 3C) indicated that the most discriminating fluxes were related to glycolysis (pgi, pgk, eno, pdh), the PPP (zwf, gnd, tk1, tk2), and the glyoxylate shunt (gs1, gs2).

### 2.6. Glucose Fluxotypes of ΔybjP and ΔydcS Strains

**Figure 4:**
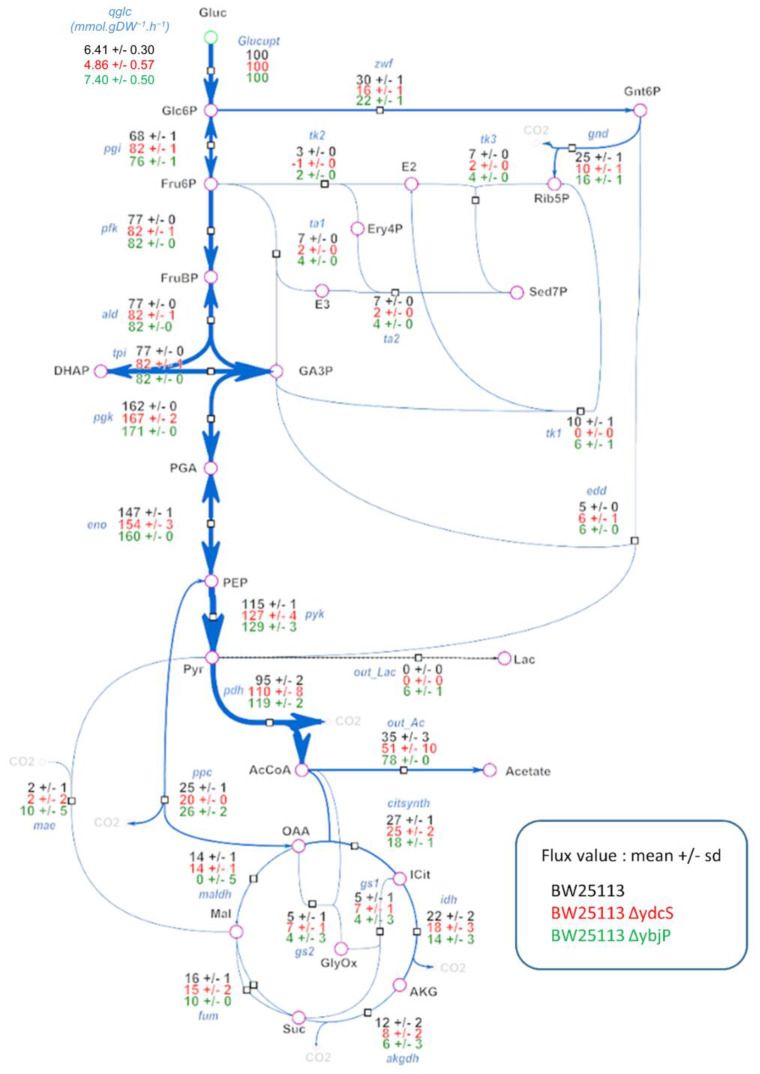
Distribution of metabolic fluxes in *Δ*ydcS and *Δ*ybjP strains. A: Intracellular flux distributions in the two mutant strains and comparison with the WT strain. All fluxes are expressed relative to the glucose uptake rate, set arbitrarily to 100 for each strain. The absolute glucose uptake rates (mmol.gDW^−1^·h^−1^) of the three strains are shown in the top left of the figure.

The glucose fluxotypes of the *ΔybjP* and *ΔydcS* strains are shown in Figure 4. Consistent with the PCA data, significant differences were observed between the two strains and the wild type strain, specifically that both strains had higher glycolytic flux than the wild type. In the *ΔybjP* and *ΔydcS* strains, 82%, and 76% of glucose was metabolized through pgi, respectively, against 68% in the wild type strain. (i.e., pgi flux in the *ΔydcS* strain). As a result, the two strains had decreased fluxes through the oxidative branch of the PPP (i.e., zwf and gnd), while the flux through the ED pathway (i.e., EDD) was stable. This trend was even stronger in the *ΔydcS* strain, for which the data also showed partial reversal of the non-oxidative branch of the PPP (tk1 and tk2 reactions), probably to compensate for the decreased production of pentose-5-P, a key precursor for major biomass components. These data suggest that the products of the *ybjP* and *ydcS* genes are either directly or indirectly involved in the control of flux in the PPP pathway.

Significant alterations of fluxes in the Tricarboxylic Acid Cycle (TCA) cycle and related pathways were observed in the two mutant strains compared to the wild-type strain. Pyruvate dehydrogenase flux was significantly increased in the *ΔydcS* strain, and even more so in the *ΔybjP* strain, reaching 119% against 95% in the wild type. This was not accompanied by increased flux through the TCA cycle, but by increased acetate excretion, as well as increased flux through the glyoxylate shunt. Lactate production was observed furthermore in the *ΔybjP* strain, and this strain had no flux through malate dehydrogenase (i.e., maldh) and increased flux through malic enzyme (i.e., mae). Overall, these data highlight a rerouting of metabolic fluxes around the acetyl-CoA node mainly resulting in the diversion of glycolytic flux from oxidative metabolism to acetate excretion in both strains. 

### 2.7. Cofactor Usage in the ΔybjP and ΔydcS Strains

**Figure 5:**
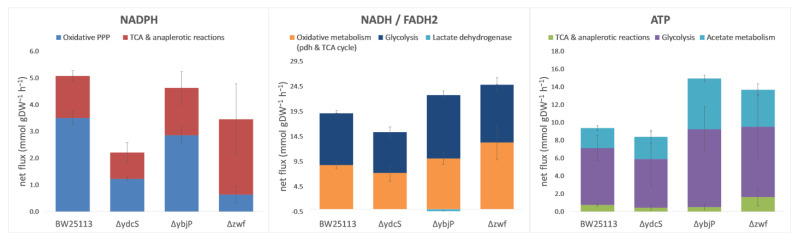
Production of NADPH, NADH/FADH_2_ and ATP in the central carbon metabolism. The rates were calculated from the mean growth rates and flux distributions, and are expressed in absolute values (mmol.gDW^−1^·h ^−1^) ± SD. Calculations are described in the materials and methods.

The changes in carbon fluxes were associated with changes in the supply of adenosine-triphosphate (ATP) and redox cofactors by the central carbon metabolism (Figure 5). The *ΔydcS* strain produced ATP at a slightly lower rate than the WT strain did. Its redox status was significantly impaired. In particular, reduced Nicotinamide Di-Nucleotide Phosphate (NADPH) production was drastically decreased, due to the much lower flux through the PPP and the absence of compensation via the TCA cycle or malic enzyme. Because this strain grows roughly as fast as the WT strain, hence, has similar biosynthetic requirements, it is likely that the reduced production of NADPH via carbon metabolism (Figure 5) is compensated by increased transhydrogenase activity [37]. The *ΔybjP* strain had different ATP and redox profiles. First, this strain produces ATP faster than the WT strain due to the significantly increased fluxes through glycolysis and acetate metabolism. Interestingly, the same ATP production profile was observed in the *Δzwf* strain (Figure 5), but the latter was significantly impaired for NADPH production, while the *ΔybjP* strain produced NADPH at only a slightly lower rate than the WT strain. This reflects a smaller reduction in PPP flux and an increase in malic enzyme flux, which accounted for 16% of CCM-derived NADPH production in this strain compared to 4% in the WT strain. The latter increase might be the result of a mechanism used to compensate for the decreased NADPH production via the oxidative PPP. In addition, NADH production was significantly higher in the *ΔybjP* mutant than in the WT strain, mainly due to a large increase in glycolytic production of NADH. This resulted in the production of lactate, suggesting a global imbalance in redox metabolism in this strain.

### 2.8. Scope and Quality of High-Throughput Fuxomics Investigations 

Given that a specific objective of this work was to deploy high-resolution fluxomics in a HT manner, we evaluated the quality of the fluxome data, obtained here, in comparison with existing data in the literature. High-throughput investigations must find the best compromise between throughput and information level (and quality). Therefore, the volume and quality of the flux data have to be appreciated in terms of the actual size of the investigation, i.e., the total number of flux maps generated. To this aim, we used the four criteria defined below:

(i) *fluxome resolution*, which corresponds to the total number of fluxes that can be calculated from the experimental dataset, and is a measure of the coverage of the flux space in the investigated metabolic network. This resolution is generally low (e.g., 23) in high-throughput studies, and high (71–84) in low-throughput ones (Table 1). In this work, we were able to measure 94 fluxes per measured fluxotype, which is the highest fluxome resolution value among all ^13^C-fluxomics studies reported in Table 1.
(ii) *isotopic resolutive power* of the flux map, defined as the ratio between the number of isotopic data and the number of fluxes calculated in the network. This index is assumed to reflect data redundancy in the flux estimates, hence, higher precision in the calculated flux values. Low-throughput investigations of the *E. coli* fluxome have isotopic resolutive power in the range of 1 to 8 (Table 1), though very high isotopic resolutive power (up to 17.6), has been obtained by performing multiple (e.g., parallel) labelling experiments to calculate a single flux map [38,39,40,41]. In the present work, the isotopic resolutive power of the flux maps was 1.13 (106 isotopic data for 94 fluxes), which is comparable to the values obtained in low-throughput investigations (Table 1).
(iii) *total flux dimension*, which is equal to the number of fluxotypes multiplied by fluxome resolution, and is an indication of the scale of the fluxomics investigation. In the literature, this index ranges between 71 and 4370 (Table 1). In this study, the total flux dimension index was 18,612 (94 fluxes × 198 fluxotypes), which reflects both the high-throughput and high-resolution character of the analysis. Interestingly, the number of fluxotypes, generated in this study, is similar to the number reported by Haverkorn van Rijsewijk et al. [33] (198 versus 190, respectively) but the total flux dimension of this work is higher (18612 versus 4370, respectively) because of the higher number of fluxes per fluxotype (94 veresus 23, respectively).
(iv) *overall flux precision* index, which is the median RSD of all (free) fluxes across the entire flux dataset. Only free fluxes are considered because the others, e.g., most biosynthetic fluxes are determined or given as constraints to the model. The average value of this index was 32% in the present study, compared with 0.4–23% in low-throughput studies, and 14–253% in HT studies (Table 1). The value, obtained in this work, appears to strike a good compromise between throughput and resolution. Moreover, when measured for specific pathways (*overall pathway-specific precision*), it appears that the level of precision achieved for most fluxes is drastically improved, notably for those in the glycolysis, PPP and ED pathways.

## 3. Discussion

This work introduces significant improvements in the parallelization, automation and implementation of fluxomics in large-scale investigations. Altogether, these improvements offer significant gains in throughput, robustness, and data quality, while retaining a high level of biological information by collecting high resolution fluxomics data. This optimized complete HT fluxomics workflow allowed 198 isotopically-resolved flux maps to be generated in a total (effective) experimental time of less than 33 days. This required a total human time of 194 h, or about 1 h per flux map. In the context of high-resolution fluxomics, the working volume of the bioreactors, 15 mL, seems an appropriate compromise between the low volumes required for HT investigations, and culture volumes, which are large enough for detailed biological or biochemical analysis. Indeed, this working volume allowed us to systematically collect all relevant physiological and biochemical data in a single bioreactor under fully controlled growth conditions, thereby decreasing biological variability and increasing data consistency. Further applications of this workflow to other experimental designs, e.g., parallel labeling experiments, other questions or other biological models (bacterial communities, eukaryotic cells, etc.) should be facilitated by the flexibility of the setup, which is amenable to a variety of sample types (medium, cells, total broth), sampling methods, metabolite extraction processes (intra- or extra-cellular metabolites, biomass), and analytical techniques (basically any targeted/untargeted LC/GC-MS method or NMR sequence). This flexibility allows metabolic networks of various size and complexity, as well as other biological models, to be investigated. There is still scope for improvement in almost all stages of the workflow—from sampling to flux calculations and sensitivity analysis—to increase throughput and reduce experimental times and human involvement. A major challenge in the application of HT fluxomics is the level of precision on flux data that can be achieved at high or very high throughput. In this regard, the capability of the robotic platform to enable a high(er) number of biological replicate experiments to be carried out certainly contributes to increase the reliability of the data. 

^13^C-Fluxomics encompasses different approaches depending on the number of isotopic data collected and number of fluxes calculated [21,23,24]. These approaches include *isotopic profiling* (i.e., the statistical analysis of large isotopic datasets to discriminate variants), *targeted 13C-fluxomics* (in which a limited number of isotopic data are collected to measure fluxes in a few reactions or pathways), and *global ^13^C-fluxomics* (the measurement of fluxes across complete metabolic networks from large isotopic datasets). The former two approaches are low-resolution methods because they provide no (isotopic profiling), or limited (targeted fluxomics), flux information, though they are fully relevant for their respective purposes. High-resolution fluxomics refer to global ^13^C-fluxomics, but the number of measured fluxes can vary significantly between studies (Table 1). HT fluxomics approaches have been discussed, in terms of methodological challenges and biological value, but so far, there has been no method proposed to evaluate the actual scale and quality of HT fluxomics investigations. This work introduces novel metrics for the actual scale and quality of HT fluxomics investigations. At the level of individual fluxotypes, key features of these studies, include the total number of calculated fluxes (*fluxome resolution*), the number of isotopic data, collected with respect to the number of fluxes to be measured (*isotopic resolutive power*), and the resulting precision in the calculated flux values (confidence intervals on flux values). At the level of the complete HT fluxomics investigation, three indices are introduced to measure the actual scale (*total flux dimension*) and quality (*overall flux precision* and *overall pathway-specific precision*) of HT fluxomics investigations. Since HT studies aim to find the best compromise between throughput and information content (and quality), the number and quality of the resulting flux data have to be appreciated, in terms of the actual size of the investigation, i.e., the total number of flux maps generated. HT fluxomics investigations are characterized by high *total flux dimension,* low isotopic resolutive power and low flux precision [22]. On the contrary, high-resolution fluxomics are characterized by low total flux dimensions, high isotopic resolutive power and high flux precision [39,40,41]. Finally, methods developed for the large-scale application of high-resolution fluxomics, such as the one described here, should aim to achieve good isotopic resolutive power and flux precision with high total flux dimension [33]. Indeed, we could apply High-resolution fluxomics (fluxome resolution of 94) at a high-throughput level (198 fluxotypes collected throughout the study) without loss of quality. It is important to note that these metrics are objective measures of the size and characteristics of fluxomics investigations; they do not quantify the biological value of the data.

The main objective of this work was to investigate whether the deletion of y-genes led to any changes in the distribution of metabolic fluxes in the central metabolism, i.e., in glucose metabolic fluxotypes. Deletion of the part of the y-ome, considered in this study, was associated with an overall tendency toward slower growth, but for the vast majority of the 180 strains investigated, the distribution of metabolic fluxes was remarkably similar to the one measured for the WT strain. Given that *E. coli* expressed the products of all the investigated y-genes during growth on glucose, there are several possible explanations for these results. (i) The protein is expressed, but is not functional under the considered culture conditions, hence, its absence has no impact in the deletion mutant. (ii) The protein has a functional role under these culture conditions, but this function is not related to metabolism, hence, its absence does not result in a metabolic phenotype. (iii) The protein has a metabolic function, as an enzyme or as a metabolic regulator in these culture conditions. In this case, the absence of a particular flux phenotype can be explained by the gene product having a quantitatively minor role, at least for the part of metabolism that was investigated, or by the onset of compensatory mechanisms that efficiently counterbalance the effects of the gene product’s absence. Further investigations, including on fluxotypes under other experimental conditions are required to answer these questions. However, regardless of the specific mechanisms involved for each y-gene, the data reported here highlight the robustness of *E. coli*’s central metabolism to the absence of all these gene products, whatever their roles, and to the potential impairments caused by their absence. 

In this work, we were able to provide highly detailed functional information on the metabolic impact of y-gene deletion. Two strains, *ΔybjP* and *ΔydcS* showed interesting glucose fluxotypes, indicating that the products of the genes are involved in metabolism during growth on glucose as sole carbon source. Information on these genes is scarce and elusive. The gene *ydcS* was annotated as a putative putrescine ATP-binding Cassette transporter in several databases and has also been proposed to encode poly-3-hydroxybutyrate synthase activity [42]. The gene *ybjP* has been predicted to be a lipoprotein [43]. Interestingly, the metabolic impact of both these genes is global, since most of the pathways of the central carbon metabolism are affected. Indeed, both mutant strains show significant perturbations in the PPP and in the TCA cycle and related pathways. The deletions are also both associated with energy and redox status modifications, but with opposite consequences. The *ΔybjP* strain has significantly impaired ATP production and reduced cofactors within the central carbon metabolism, while the *ΔybjP* has higher production levels than observed in the WT strain. The fact that the metabolic effects are so wide-ranging suggests that the products of these genes affect central metabolic parameters, such as the redox status or that they are global regulators of metabolism, either directly or indirectly. The dispensability of the two y-genes during growth on various carbon sources has recently been documented [12]. The *ΔydcS* strain shows reduced growth on certain sugars (mainly mannose, glucosamine, fructose) and two organic acids (alpha-ketoglutarate and acetate). The *ΔybjP* strain shows reduced growth on a few carbon sources, mainly including pyruvate, ribose, sorbitol and galacturonate. Deletion of these y-genes has no effect [12] or only a slight effect (this work) on growth with glucose as sole carbon source, in spite of significant metabolic changes in the two mutant strains. These observations emphasize that gene dispensability does not mean the gene lacks a metabolic function, but rather that the metabolism has successfully adapted to the absence of the gene product [4]. This observation stresses the need to measure the actual metabolic phenotype of mutants, even if the gene deletion is silent in terms of growth. In this context, methods like High-resolution fluxotyping are essential in achieving both the throughput and level of metabolic information required to investigate the role of y-genes in the metabolism of diverse carbon sources.

To conclude, this work shows that high-resolution fluxomics is amenable to high-throughput investigations and can provide detailed information on metabolic phenotypes. Our results also highlight how it can reveal the metabolic impact of gene deletion, even if the gene has no known function or growth phenotype.

## 4. Materials and Methods 

### 4.1. Bacteria Strains and Cultivation Conditions

*E. coli* BW 25,113 and the derived strains used in this study (listed in Supplementary data 1) were taken from the Keio collection (Baba et al., 2006). One hundred and eighty y-gene mutants were selected from the original glycerol stock of 3985 single-gene deletion mutants available in this collection (*E. coli* BW 25,113 strains mutants; more details directly in paragraph 2.1 of the paper). The *E. coli* strain MG1655 was also used. All the selected strains were first cultivated overnight in Luria-Bertani (LB) medium (10 g/L tryptone, 5 g/L yeast extract and 10 g/L NaCl) with kanamycine (25 ug/mL) at 37 °C and then stored in glycerol stocks. 

To perform the experiments, the glycerol stocks of relevant strains were used to inoculate liquid LB medium in microplates. The LB cultures were used to inoculate precultures in minimal synthetic medium containing 17.4 g·L^−1^ Na_2_HPO_4_, 12H_2_O, 3.03 g·L^−1^ KH_2_PO_4_, 0.51 g·L^−1^ NaCl, 2.04 g·L^−1^ NH_4_Cl, 0.49 g·L^−1^ MgSO_4_, 4.38 mg·L^−1^ CaCl_2_, 15 mg·L^−1^ Na_2_EDTA 2H_2_O, 4.5 mg/L ZnSO_4_ 7H_2_O, 0.3 mg·L^−1^ CoCl_2_ 6H_2_O, 1 mg·L^−1^ MnCl_2_ 4H_2_O, 1 mg·L^−1^ H_3_BO_3_, 0.4 mg·L^−1^ Na_2_MoO_4_ 2H_2_O, 3 mg·L^−1^ FeSO_4_ 7H_2_O, 0.3 mg·L^−1^ CuSO_4_ 5H_2_O, 0.1 g·L^−1^ thiamine and 3 g·L^−1^ glucose. For the ^13^C-labeling experiments, unlabeled glucose was replaced by the same concentration of a mixture of 80% [1-^13^C_1_]-D-glucose + 20% [U-^13^C_6_]-D-glucose. To minimize sources of unlabeled carbon atoms from the first cultivation steps in subsequent experiments, the cells were inoculated at a starting OD between 0.08 and 0.12.

### 4.2. Robotic Platforms for Culture, Sampling and Sample Preparation

Two robotic platforms were used to parallelize the cell cultures, ^13^C-labelling experiments, sampling of labeled metabolites, and sample preparation for NMR and MS analyses. The first system allowed 48 ^13^C-labelling experiments to be performed automatically in parallel in 15 mL bioreactors under controlled growth conditions, with automated collection of labelled samples (of biomass or culture medium) at defined culture times or optical densities. The robot and its operation are described in detail by Heux et al. [22]. A second robotic workstation (Freedom EVO 200, Tecan, Männedorf, Switzerland) was designed and assembled to fully automate and parallelize the final preparation of labeled biological samples for the analysis of isotopic profiles by NMR or mass spectrometry. This device was used to handle the different containers used (NMR tubes, vials, multiwell-plates, etc.), in order to perform dilutions, add standards, take aliquots, and manage the samples.

The ^13^C-labeling experiments were carried out in batches of 48 parallel cell cultures using the two robotic platforms described above. The input label was optimized for *E. coli*’s metabolic network using IsoDesign (1.2.1) [24], and consisted of a mixture of 80% [1-^13^C_1_]-D-glucose and 20% [U-^13^C_6_]-D-glucose. All cultures were performed in 15 mL reaction vessels, at 37 °C, pH = 7, a stirring speed of 2300 rpm and with 5 L·min^−1^ of compressed air fed into the culture module. Four to eight supernatant samples were collected in each reactor throughout the culture to analyze the exometabolome and to calculate the rates of substrate consumption and end-product production. The biomass was automatically sampled once the OD_600nm_ had reached 1.2, indicating that the metabolic and isotopic steady-state had been achieved: 4 mL samples of culture were pelleted by automated centrifugation (5 min/4410 g), manually hydrolyzed with 250 µL 6 N HCl for 15 h at 110 °C and washed twice in 1 mL D_2_O by rotary evaporation (Büchi Labortechnik AG, CH, Flawil, Switzerland) between each washing step. Aliquots (10 µL) of biomass hydrolysate supernatant were then collected and transferred into 96 well plates, diluted with 990 µL pure H_2_O and transferred to vials for LC-MS analysis. Samples of biomass hydrolysate supernatant (150 µL) were also mixed with 50 µL of TSP d4 (4 mM in D_2_O) and 150 µL of each mixture was then transferred into 3 mm NMR tubes. 

### 4.3. Isotopic Profiling of Proteinogenic Amino-Acids

The incorporation of ^13^C-label into proteinogenic amino acids (listed in Supplementary data 6) was analyzed by liquid chromatography–mass spectrometry, using an Ultimate 3000 HPLC system (Dionex, Sunnyvale, CA, USA) coupled to an LTQ Orbitrap Velos mass spectrometer (Thermo Fisher Scientific, Waltham, MA, USA) equipped with a heated electrospray ionization probe described in detail by Heuillet et al., 2018 [44]. Full scan HRMS analyses were performed in positive FTMS mode at a resolution of 60,000 (at 400 *m/z*), using the following source parameters: Capillary temperature, 275 °C; source heater temperature, 250 °C; sheath gas flow rate, 45 a.u. (arbitrary unit); auxiliary gas flow rate, 20 a.u.; S-Lens RF level, 40%; source voltage, 5 kV. Metabolites were identified by extracting the exact mass with a tolerance of 5 ppm. The raw MS isotopic profiles of proteinogenic amino acids were then quantified using Tracefinder (Thermo Fisher Scientific, Waltham, MA, USA). The isotopic profiles (Carbon Isotopologue Distributions) were obtained after correcting for natural isotopic abundances using IsoCor 1.2 [27,28] (https://github.com/MetaSys-LISBP/IsoCor (accessed on 20 May 2020)). The raw MS data are available from Metabolights (accession number MTBLS2188).

### 4.4. NMR Analysis of Extracellular Medium

Culture supernatants were analyzed by 1D-^1^H NMR on a Bruker Avance III 800MHz spectrometer (Bruker BioSpin, Rheinstetten, Germany) page equipped with a 5 mm CQPI cryoprobe at 280 K. To precisely quantify the extracellular compounds, the 1D-^1^H NMR data were recorded after a 30° presaturation pulse (zgpr30), with a relaxation delay of 7 s. NMR spectra were processed using Topspin 3.5pl6 (Bruker BioSpin, Rheinstetten, Germany). The raw NMR data are available from Metabolights (accession number MTBLS2188).

The rate of glucose consumption and the rates of acetate and lactate production were quantified by analyzing the four-to-eight samples collected during the cell culture period. The ^13^C labeling profiles of acetate and lactate, including positional information on label incorporation, were also measured for the flux calculations.

### 4.5. Growth Parameters

The growth parameters calculated from the experimental datasets for each culture were the growth rate, glucose uptake rate, acetate and lactate production rates and biomass yield. The growth rate was calculated from OD values measured during the culture period. The rates of glucose consumption and of acetate and lactate production were calculated from the exometabolome data. All calculations were performed using Physiofit (v0.9) [45], a software designed to determine growth parameters by fitting time-course data. The software includes a batch calculation mode, which allows calculations on large series of datasets, and which was used to calculate the growth parameters for all considered experimental conditions. The biomass yields were calculated using the conversion factor 0.378 gDW/OD600nm unit.

Extracellular fluxes were determined from the rates of disappearance (or appearance) of substrates and products, in the culture supernatants, as measured by NMR.

### 4.6. Flux Calculation and Visualization

Fluxes were calculated using the software influx_si 4.1 [30] (https://metasys.insa-toulouse.fr/software/influx/ (accessed on 20 May 2020)), including the mass balances and carbon atom transitions of the biochemical reaction network. The metabolic network contained the main pathways of *E. coli*’s central metabolism: glycolysis (EMP), the pentose phosphate pathway (PPP), the Entner-Doudoroff (ED) pathway, the tricarboxylic acid cycle (TCA), and anaplerotic reactions, the glyoxylate shunt, and the reactions for amino acid biosynthesis [32]. Intracellular fluxes were estimated from measurements of extracellular fluxes and from the ^13^C-labelling patterns of metabolites using appropriate mathematical models of glucose metabolism in *E. coli* [32]. Labeling data were collected from intracellular metabolites by IC-MS/MS and from metabolic end-products by 1D ^1^H NMR, as detailed above. The fluxes were normalized to the rate of substrate uptake, which was arbitrarily set at 100.

Metabolic fluxes were calculated for each of the 198 experimental conditions considered in the study. For each culture condition, the information required for the flux calculation (i.e., metabolic network, isotopic data, etc.) was written in a specific FTBL file [16], which was then submitted to influx_si. 

The FTBL model was converted by an influx_si module (ftbl2metxml.py) into Systems Biology Markup Language (SBML, in xml format), readable by the online application Metexplore (http://metexplore.toulouse.inra.fr (accessed on 20 May 2020)). Metexplore was used to visualize flux maps for strains of interest [46].

### 4.7. Sensitivity Analysis

Confidence intervals for the calculated fluxes were determined using a Monte-Carlo approach in which 100 independent optimization runs were performed on datasets with noise added in proportion to standard measurement errors. The isotopic data and metabolic fluxes for each independent biological replicate are provided in the Supplemental data (dataset S1).

### 4.8. Statistical Analysis of Flux Datasets

Flux maps were compared by PCA using the collaborative portal Workflow for Metabolomics (W4M) [36]. The results were visualized as boxplots and figures were generated using the software R. 

### 4.9. Meta-Data Management

The data and meta-data, generated throughout the workflow, were stored, managed, and processed as described below. 

Culture and sampling data are related to the parallel cultures of *E. coli* strains with ^13^C-labelled compounds and to the collection of samples: Settings and data from the robotic culture platform (Tecan Evo 200) were automatically and separately recorded for each of the 48 bioreactors: pH values, O2 values, OD_600nm_ values, volumes, sampling times, and tube locations and associated barcodes. The data were linked and stored in an SQL database, making them traceable to each robotic run. The most important elements of this database are listed in Supplementary data 3 (mu, batch number, batch position, biomass (mg/L) at sampling time). A log file was also automatically generated for each robotic run, detailing errors and software information.

Sample preparation data: All the steps performed on the sample preparation platform (Tecan Evo 200) were automatically recorded in an SQL database: Volumes, tube locations on the worktable, associated barcodes and sampling times. The data were visualized and previous runs re-examined using the robot’s traceability software, and a log file was automatically generated for each run with errors and software information. 

Analytical data: All mass spectrometry and NMR data were saved on a server to guarantee sample traceability. This included the raw MS data, the MS data processed with Tracefinder, the MS data processed with Isocor, the raw NMR data, the NMR data processed with TopSpin, and the NMR data processed with Physiofit. Corrected CID values, extracellular fluxes and the positional enrichment of extracellular metabolites are listed in Supplementary data 3. 

Flux calculations: All flux calculation files were stored on a server. This included the input (FTBL) files for influx_si, and the output (KVH) files. The most important elements are included in Supplementary data 3 (chi-square tests of the fits, fit cost functions and net fluxes). 

Statistical analysis and flux mapping: All the data used and generated for statistical analysis and flux mapping were stored on a server.

### 4.10. Calculation of Cofactor Production and Consumption Rates

The production rates (mmol.gDW^−1^·h^−1^) of NADPH, NADH/FADH_2_, and ATP in the central carbon metabolism were calculated as the sum of the estimated fluxes of the reactions that are expected to produce (positive value) or consume (negative value) the cofactors. Values were averaged over the biological replicates. NADPH production: Pentose-Phosphate Pathway (PPP) = glucose 6-phosphate dehydrogenase (zwf) + phosphogluconate dehydrogenase (gnd); TCA cycle = isocitrate dehydrogenase (idh); malic enzyme (mae). NADH production: glycolysis = glyceraldehyde-3-phosphate dehydrogenase (flux equal to pgk); Oxidative metabolism = pyruvate dehydrogenase (pdh) + TCA cycle enzymes, including alpha-ketoglutarate dehydrogenase (akgdh) + malate dehydrogenase (maldh); malic enzyme (mae) and biomass formation. NADH consumption: lactate dehydrogenase (out_Lac). FADH_2_ production: TCA cycle = succinate dehydrogenase (flux equal to fum). ATP production: glycolysis = phosphoglycerate kinase (pgk) + pyruvate kinase (pyk); TCA cycle = succinyl-CoA synthetase (assimilated to akgdh); acetate metabolism = acetate kinase (ack, assimilated to out_Ac). ATP consumption: glycolysis = glucose PTS system (Glucupt_U + Glucupt_1) (assuming 1 consumed PEP is equivalent to 1 consumed) + 6-phosphofructokinase (pfk); ppc (equivalent to pep carboxylase (forward flux) and pep carboxykinase (reverse flux).

## Figures and Tables

**Table 1:** Evaluation of data size and quality in HT fluxomics investigations. The overall flux precision is the median RSD (relative standard deviation) calculated for all fluxes measured in each of the studied conditions, as given in the supplementary data of the cited studies. The overall pathway precision was calculated similarly by grouping the RSDs according to the metabolic pathways considered. The flux RSDs considered in these calculations were obtained by sensitivity analysis (S.A.) or by calculating classical standard deviations on the mean values of fluxes obtained when biological replicates were produced (as in this work).

	Leighty et al., 2013 [37]	Crown et al., 2015 [38]	Long & Antoniewicz 2019 [35]	Millard et al., 2014 [30]	Long et al., 2016 [39]	Heux et al., 2014 [22]	Haverkorn van Rijsewijk et al., 2011 [31]	This Paper	
*E. coli* strains	*MG1655*	*MG1655*	*BW25113* *ΔtpiA mutant*	*MG1655*	*BW25113*	*MG1655*	*BW25113 + Keio mutants*	*MG1655 + BW25113 + keio mutants*	
Number of measured fluxotypes	1	1	1	1	1	20	190	198	
Number of different label input(s) by strain or condition	6	14	2	1	1	2	2	1	
Fluxome resolution = number of (net) fluxes by fluxotype	71	71	75	84	71	23	23	94	
Isotopic resolutive power = number of isotopic data/number of calculated fluxes	7.52	17.55	5.07	1.4	1.94	2.57	1.61	1.13	
Total flux dimension = number of fluxes per fluxotype × number of fluxotypes	71	71	75	84	142	460	4370	18,612	
Global flux precision = median RSD over the global dataset	12%	15%	7.80%	19%	23%	285%	14%	32%	
Global pathway precision = median RSD within specific pathways	S.A. (*n* = 1)	S.A. (*n* = 1)	S.A. (*n* = 1)	S.A. (*n* = 1)	S.A. (*n* = 1)	S.A. (*n* = 23)	S.A. (*n* = 23)	S.A. (*n* = 198)	biological replicates (*n* = 20)
Glycolysis	1%	2%	4.20%	3%	3.50%	553%	7%	3%	1%
ppp + edp	14%	11%	17%	131%	10%	41%	25%	24%	7%
tca + gs	11%	19%	4.90%	20%	31%	795%	19%	40%	21%
anaplerosis	122%	144%	87%	13%	1822%	7%	15%	42%	20%
output fluxes	12%	25%	4.7%	15%	42.4%	426%	0%	10%	14%

## Data Availability

Restrictions apply to the availability of these data. Data was obtained from Metabolights and are available at www.ebi.ac.uk/metabolights/MTBLS2188 (accessed on 22 April 2021) with the permission of Metabolights.

## Supplementary Materials

The following are available online at https://www.mdpi.com/article/10.3390/metabo11050271/s1, Supplementary data 1: List of the 180 *E. coli* BW 25113 strains selected for the fluxomics experiments, Supplementary data 2: Metabolic model considered in the flux calculations, Supplementary data 3: Complete dataset obtained for all the fluxomic experiments performed in the study, Supplementary data 4: Calculation of ATP and redox fluxes, Supplementary data 5: Isotopic data collected by LC HRMS and NMR.
