# Supplementary material for: Exploring the Glucose Fluxotype of the E. coli y-ome Using High-Resolution Fluxomics
Source: Metabolites. 2021 Apr 26;11(5):271. doi: 10.3390/metabo11050271 (PMC8145925; doi:10.3390/metabo11050271)
Supplement: Supplementary file 1 [file metabolites-11-00271-s001.zip › metabolites-1176208-supp-final/supdata/Supplementary_data_5_Isotopic data collected by LC HRMS and NMR.pdf]

Exploring the Glucose fluxotype of the *E. coli* y-ome using high-resolution fluxomics

Berges C., Cahoreau E. et al.

Supplementary information

List of carbon isotopologues measured by LC-MS on proteinogenic amino-acids

Metabolite	Formula	Isotopologue	$[M+H]^+$
Alanine	C3H7NO2	M0	90,0548
		M1	91,0582
		M2	92,0615
		M3	93,0649
Arginine	C6H14N4O2	M0	175,1187
		M1	176,1221
		M2	177,1254
		M3	178,1288
		M4	179,1322
		M5	180,1355
		M6	181,1389
Aspartate	C4H7NO4	M0	134,0446
		M1	135,0480

		M2	136,0513
		M3	137,0547
		M4	138,0580
Glutamate	$C_5H_9NO_4$	M0	148,0603
		M1	149,0637
		M2	150,0670
		M3	151,0704
		M4	152,0737
		M5	153,0771
Glycine	$C_2H_5NO_2$	M0	76,0393
		M1	77,0426
		M2	78,0460
Histidine	$C_6H_9N_3O_2$	M0	156,0766
		M1	157,0799
		M2	158,0833
		M3	159,0866
		M4	160,0900
		M5	161,0933
		M6	162,0967

Isoleucine	C6H13NO2	M0	132,1018
		M1	133,1051
		M2	134,1085
		M3	135,1118
		M4	136,1152
		M5	137,1185
		M6	138,1219
Leucine	C6H13NO2	M0	132,1018
		M1	133,1051
		M2	134,1085
		M3	135,1118
		M4	136,1152
		M5	137,1185
		M6	138,1219
Lysine	C6H14N2O2	M0	147,1127
		M1	148,1161
		M2	149,1194
		M3	150,1228
		M4	151,1261

		M5	152,1295
		M6	153,1328
Methionine	$C5H11NO2S$	M0	150,0582
		M1	151,0616
		M2	152,0649
		M3	153,0683
		M4	154,0716
		M5	155,0750
Phenylalanine	$C9H11NO2$	M0	166,0859
		M1	167,0893
		M2	168,0926
		M3	169,0960
		M4	170,0993
		M5	171,1027
		M6	172,1061
		M7	173,1094
		M8	174,1128
		M9	175,1161
Proline	$C5H9NO2$	M0	116,0703

		M1	117,0737
		M2	118,0770
		M3	119,0804
		M4	120,0837
		M5	121,0871
Serine	$C_3H_7NO_3$	M0	106,0498
		M1	107,0532
		M2	108,0565
		M3	109,0599
Threonine	$C_4H_9NO_3$	M0	120,0654
		M1	121,0688
		M2	122,0721
		M3	123,0755
		M4	124,0788
Tyrosine	$C_9H_{11}NO_3$	M0	182,0810
		M1	183,0844
		M2	184,0877
		M3	185,0911
		M4	186,0944

		M5	187,0978
		M6	188,1011
		M7	189,1045
		M8	190,1079
		M9	191,1112
Valine	C5H11NO2	M0	118,0862
		M1	119,0896
		M2	120,0929
		M3	121,0963
		M4	122,0996
		M5	123,1030

List of 1H-NMR signals exploited for the quantification and isotopic profiling of medium compounds

**Metabolite**	**1H Spin system**	**Chemical shift**	**Scalar coupling (J_{CH})**
Glucose	H1a (13C)	5.343 ppm	169.6 Hz
	H1b (13C)	4.549 ppm	162 Hz
Acetate	H3 (13C)	2.003 ppm	127.1 Hz
	H3 (12C)	1.925 ppm	
	H3 (13C)	1.844 ppm	
Lactate	H3 (13C)	1.412 ppm	128.4 Hz
	H3 (12C)	1.334 ppm	
	H3 (13C)	1.252 ppm	
